# En bloc excision and autogenous fibular reconstruction for aggressive giant cell tumor of distal radius: a report of 12 cases and review of literature

**DOI:** 10.1186/1749-799X-6-14

**Published:** 2011-03-08

**Authors:** Raghav Saini, Kamal Bali, Vikas Bachhal, Aditya K Mootha, Mandeep S Dhillon, Shivinder S Gill

**Affiliations:** 1 Deptt of Orthopaedics, Postgraduate Institute of Medical Education and Research (PGIMER), Chandigarh, India; 2 Vice Chancellor, Baba Farid University, Faridkot, India

## Abstract

**Introduction:**

Giant cell tumor (GCT) of distal radius follows a comparatively aggressive behaviour. Wide excision is the management of choice, but this creates a defect at the distal end of radius. The preffered modalities for reconstruction of such a defect include vascularized/non-vascularized bone graft, osteoarticular allografts and custom-made prosthesis. We here present our experience with wide resection and non-vascularised autogenous fibula grafting for GCT of distal radius.

**Materials and methods:**

Twelve patients with a mean age of 34.7 years (21-43 years) with Campanacci Grade II/III GCT of distal radius were managed with wide excision of tumor and reconstruction with ipsilateral nonvascularised fibula, fixed with small fragment plate to the remnant of the radius. Primary autogenous iliac crest grafting was done at the fibuloradial junction in all the patients.

**Results:**

Mean follow up period was 5.8 years (8.2-3.7 years). Average time for union at fibuloradial junction was 33 weeks (14-69 weeks). Mean grip strength of involved side was 71% (42-86%). The average range of movements were 52° forearm supination, 37° forearm pronation, 42° of wrist palmerflexion and 31° of wrist dorsiflexion with combined movements of 162°. Overall revised musculoskeletal tumor society (MSTS) score averaged 91.38% (76.67-93.33%) with five excellent, four good and three satisfactory results. There were no cases with graft related complications or deep infections, 3 cases with wrist subluxation, 2 cases with non union (which subsequently united with bone grafting) and 1 case of tumor recurrence.

**Conclusion:**

Although complication rate is high, autogenous non-vascularised fibular autograft reconstruction of distal radius can be considered as a reasonable option after en bloc excision of Grade II/III GCT.

## Introduction

Giant cell tumor is a benign aggressive bone tumor of obscure origin presenting in 3^rd ^and 4^th ^decade of life, and carries a definite female preponderance [1]. After distal femur and proximal tibia, distal radius happens to be the most common site of occurrence for GCT [1,2]. This site has a further distinction of having more aggressive behaviour of GCT with higher chances of recurrences and malignant transformation [3,4]. Treatment options for GCT at this site include curettage with bone grafting or cementing, en bloc excision and reconstruction with non vascular or vascular fibular autograft, osteoarticular allograft, ulnar translocation, or endoprosthesis [5-14]. Although amputation would seem likely to be curative, it is seldom warranted in a tumor that rarely metastasizes.

The recurrence rate for primary treatment of GCT is relatively higher for curettage or extended curettage as compared to en bloc excision, making latter a more suitable and reliable option in cases showing aggressive lesions which so often is the case in distal radius [2,3,8,15,16]. Although providing the best chance of cure from GCT, en bloc excision of distal radius presents complex reconstructive problems [16-21]. Reconstruction of wrist after en bloc excision of distal radius is a challenging task. Most patients are young active adults demanding cosmetically acceptable and functionally adequate wrist. We have routinely used ipsilateral non vascularised fibular autograft for reconstructing distal radius and present here our experience with this procedure.

## Materials and methods

On retrospective search of our hospital records, we found 15 cases of GCT distal radius operated with non vascularised fibular autograft reconstruction of distal radius at our institute during a period from 2002 to 2007 and we were able to follow 12 of those cases. Patients were classified according to Campanacci's radiological grading method consisting of three grades [22]. Grade I tumors had a well-defined border of a thin rim of mature bone and bony cortex was intact. Grade II lesions had relatively well-defined margins but there was no radio-opaque cortical rim. Grade III was designated to the lesions with fuzzy borders, suggesting a rapid, and possibly a permeative, growth of the tumor.

All patients with grade I tumors are treated with extended curettage at our institute in a hope to avoid more radical surgery and this series includes only grade II and III treated with autograft reconstruction. Grade III tumors have been uniformly treated by autograft reconstruction in our institute. However, the decision type of operative intervention (extended curettage vs resection/reconstruction) in grade II tumors was based on individual case with with one of the important consideration being the subcortical bone stock likely to be available after curettage.

**Figure 1:**
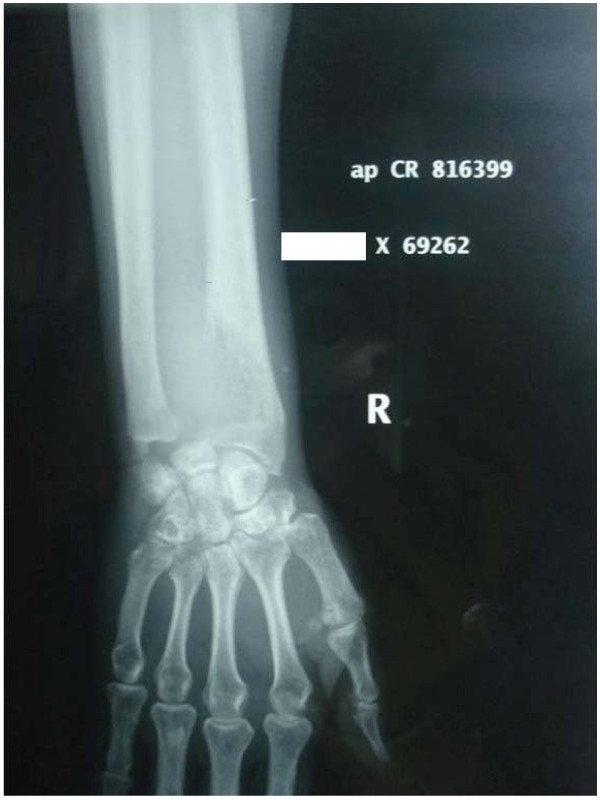
**Preoperative radiograph showing GCT of the distal radius**.

Autograft reconstruction was the index surgery in 10 of the 12 cases and rest 2 were cases of recurrent GCT initially treated with extended curettage and bone cementing for these grade II tumors. Of the 10 primary cases, initial preoperative biopsy confirmation of GCT was done in 3 cases, all of which had a grade III tumor and there was a suspicion of a malignant neoplasm on account of aggressive radiographic picture. In remaining 7 cases, an intraoperative frozen section confirmed the benign nature of neoplasm before surgery proceeded to autograft reconstruction. All patients were evaluated preoperatively with plain radiograph (Figure 1) and MRI of involved wrist and with plain radiograph of chest. Serum calcium, phosphorus and alkaline phosphatase were also determined to rule out hyperparathyroidism.

### Procedure

**Figure 2:**
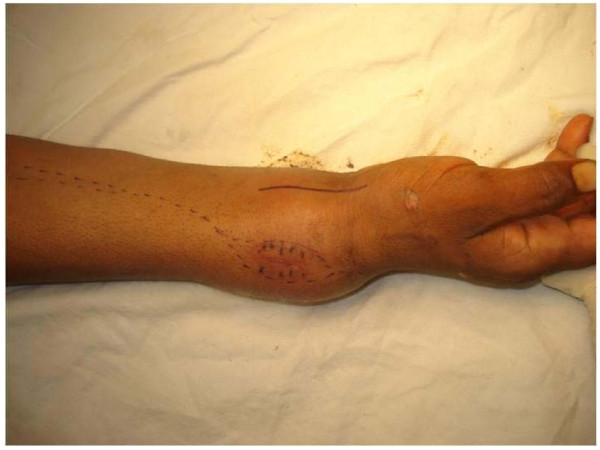
**Clinical picture showing the biopsy scar and incision outline highlighting how the biopsy tract has to be carefully excised in this patient**.

Patients were operated under general anaesthesia and ipsilateral leg, arm and iliac crest were prepped and draped appropriately. A pneumatic tourniquet was used at both surgical sites. Surgical approach chosen for distal radius depended on site of radiographic thinning or breach of cortical bone. Thus 9 cases were operated from dorsal exposure and rest 3 from volar. Biopsy tract, if present, was taken in the inital incision (Figure 2). Bone was resected at a level determined preoperatively based on extent of bone involvement on MRI plus a safe margin of 3-5 cm. On an average 10.5 cm (8-13 cm) of bone was resected. Dissection remained extraperiosteal at all time in order to avoid spillage of tumourous tissue and a soft tissue cuff was excised along with the tumor taking care not to damage neurovascular structures. After excision, tumor bed was routinely treated with 5% phenol and 3% hydrogen peroxide to take care of the inadvertent spillage, if any. We tried to avoid resecting all of the radiocarpal ligaments, if not involved, as these were later repaired to ligaments attached to proximal fibula to form a stable wrist joint.

Ipsilateral fibula was approached from standard direct lateral approach after identifying and carefully protecting the common peroneal nerve. We routinely carried dissection into the distal third of thigh to identify peroneal nerve as it is easier and safer to do so at this site where the nerve runs along the posterior border of biceps femoris. Fibula was sectioned at desired length depending on the defect created in forearm after tumor resection. We routinely obtained 3-5 mm extra length of fibula to cover for compression at radio fibular junction and error in taking measurements. While freeing the proximal tibiofibular articulation some length of lateral ligaments attached to fibular head were retained with the graft. After resection, lateral collateral ligament and biceps femoris tendon were reattached to tibia through drill holes made for this purpose. Haemostasis was achieved before closing the wound over a suction drain.

**Figure 3:**
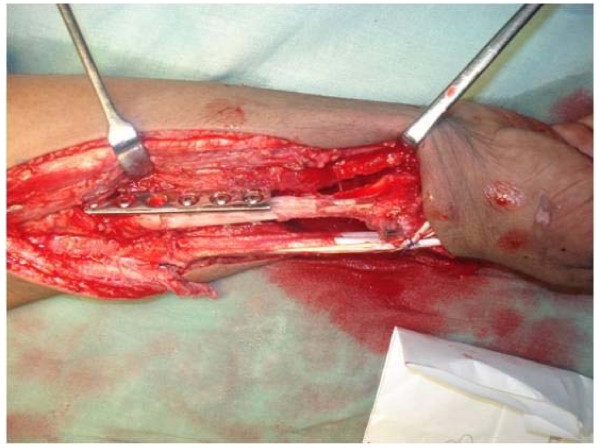
**Intraoperative picture showing the fixation of harvested fibular autograft using small fragment LCDCP**.

**Figure 4:**
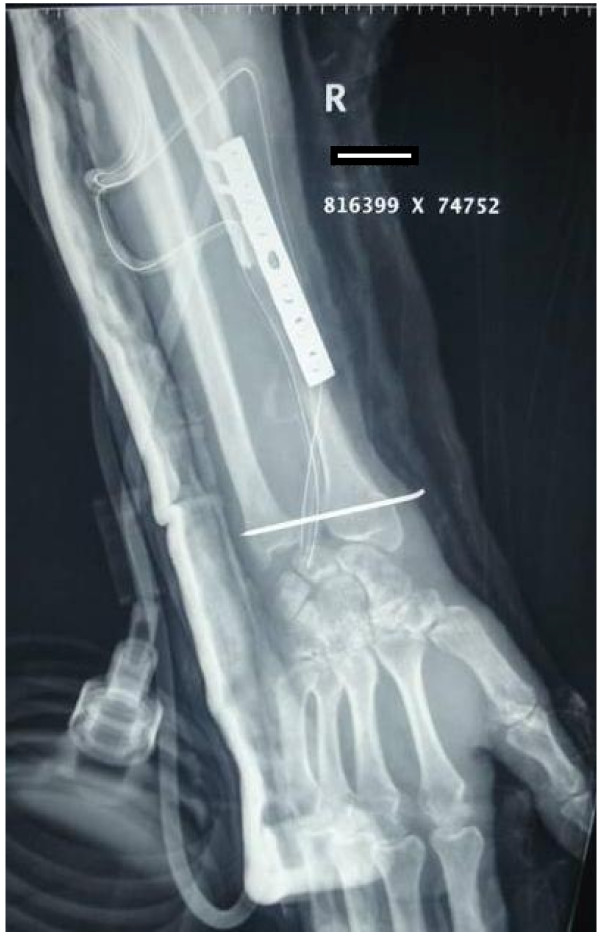
**Immediate post operative radiograph of the same patient**.

Newly harvested fibular graft was placed in ipsilateral forearm and radiocarpal ligaments were repaired to lateral collateral ligament. After reduction of newly formed fibula carpal joint, fibular diaphysis was reduced to remaining proximal radius. At this moment, tension in soft tissue and fibuloulnar articulation was determined and appropriate adjustments were made in graft length if deemed necessary. We aimed to maintain distal extent of fibular graft about 5 mm distal to tip of ulnar styloid. Graft was then secured to radius using a 6 or 7 hole 3.5 mm small fragment Low contact dynamic compression plate (LCDCP) (Figure 3). A k wire was then passed from fibula to ulna to stabilize fibuloulnar articulation (Figure 4). Another k wire was used to stabilize fibulocarpal joint, if thought necessary but not routinely. An iliac crest bone graft from ipsilateral side was routinely taken and applied at fibuloradial junction. After careful haemostasis, wound was closed over a suction drain and an above elbow slab was applied.

**Figure 5:**
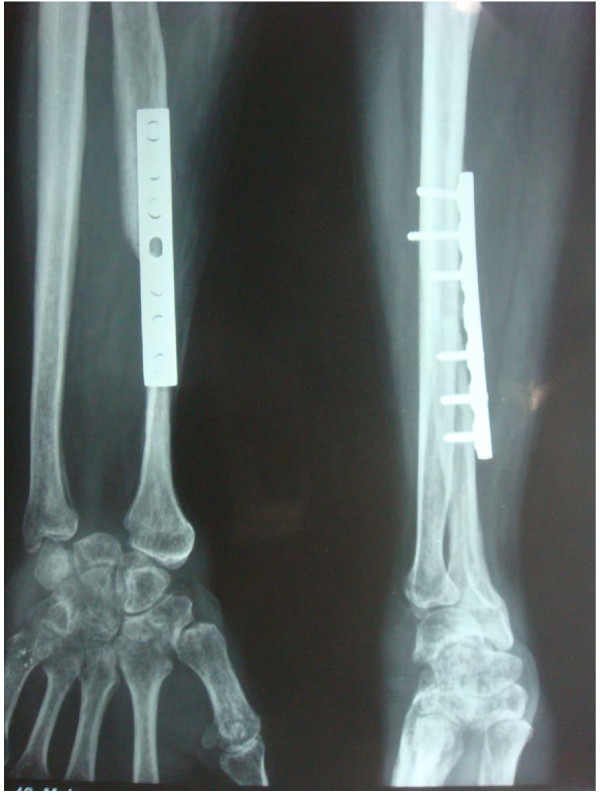
**Follow up radiographs showing union of the fibular graft with the radius**.

Full weight bearing was allowed as tolerated. Above elbow slab was continued for two months and then K wires were removed and a functional brace was applied thus allowing elbow mobilisation. After 3 months gentle active and assisted wrist exercises were started and gradually increased in intensity depending on tolerance and progress. No heavy activity was allowed for a full one year. At 3 months, plain radiographs of forearm were repeated to see for union, recurrence of tumor or graft related complications (Figure 5). After first year, follow up was at 3 monthly intervals for one year and 6 monthly in 3^rd ^year. Thereafter patients were evaluated annually till latest follow up. A dynamometer was employed to measure grip strength and compared to opposite normal side. Similarly, a goniometer was used to measure range of movement and compared to opposite side. At most recent follow up, functional results were reported using the revised musculoskeletal tumor society score which scores patients based on factors (pain, functional activities, and emotional acceptance) pertinent to patient as a whole and factors specific to either upper limb (positioning of hand, manual dexterity, and lifting ability) or the lower limb [23]. Results were established as excellent for MSTS score > 90%, good for 80-90%, satisfactory for 60-80% and poor for ≤ 60% score. We further downgraded patient's result by one tier if they developed any significant complication.

## Results

Table 1 and Table 2 summarises the patient profile and the results.

**Table 1:** Patients profile and results

**S.No**.	Age in years	Grade	Sex	Follow Up (years)	Supination (degrees)	Pronation (degrees)	Palmer Flexion (degrees)	Dorsi Flexion (degrees)	Combined range of motion as percentage of opposite side (operated side/normal side in degrees)	Grip Strength as percentage of normal side (operated side/normal side in Kg)	**MSTS Score**^@^	**Complications**^$^	Result*
1	34	II	F	4.8	60	25	40	20	55% (145/265)	75% (24/32)	93.33%	WI	G

2	21	II	M	7.1	75	40	55	30	78% (200/255)	86% (43/50)	93.33%		E

3	39	III	M	4.6	50	35	30	45	67% (160/240)	78% (42/54)	93.33%	WI	G

4	43	II	M	8.2	45	60	45	25	71% (175/245)	64% (30/47)	93.33%	NU	G

5	30	III	M	4.2	65	30	40	50	70% (185/265)	74% (40/54)	93.33%		E

6	36	III	F	5.5	35	25	20	15	34% (95/280)	67% (16/24)	90%	WI	S

7	33	II	M	3.7	40	55	70	30	75% (195/260)	85% (40/47)	93.33%		E

8	37	III	F	6.5	60	35	40	55	76% (190/250)	78% (21/27)	93.33%		E

9	29	III	F	7.2	55	50	35	40	71% (180/255)	79% (26/33)	93.33%	NU	G

10	35	III	M	6.3	70	30	45	15	64% (160/250)	83% (29/35)	90%	R	S

11	38	II	M	7.5	45	40	60	40	73% (185/255)	42% (16/38)	93.33%		E

12	41	III	F	4.0	25	20	25	10	29% (80/280)	45% (17/38)	76.67%		S

Mean				5.8	52	37	42	31	64% (162/258)	71% (29/40)	91.38%		

@ MSTS score is calculated as percentage of the maximum possible score of 30.

$ WI = Wrist instability, NU = Non-union, R = Recurrence.

* Results were downgraded one tier in patients having complications. E = excellent, G = good, S = satisfactory.

**Table 2:** Revised Musculoskeletal Tumor Society Score of Individual patients

**S.No**.	Pain	Function	Emotional Acceptability	Hand Positioning	Dexterity	Lifting Ability	Total	MSTS score^@^
1	3	5	5	5	5	5	28	93.33

2	5	4	5	5	5	4	28	93.33

3	3	5	5	5	5	5	28	93.33

4	4	5	5	4	5	5	28	93.33

5	5	4	5	5	5	4	28	93.33

6	3	5	4	5	5	5	27	90

7	4	5	5	4	5	5	28	93.33

8	4	5	5	5	5	4	28	93.33

9	5	5	4	5	4	5	28	93.33

10	4	4	5	4	5	5	27	90

11	4	5	5	5	5	4	28	93.33

12	3	4	5	3	4	4	23	76.67

Mean							27.42	91.38

@ MSTS score is calculated as percentage of the maximum possible score of 30.

Of the 12 patients analysed, there were 7 males and 5 females with 8 left sided and 4 right sided involvement of distal radius. The mean age of patients included in analysis was 34.7 years (21-43 years). There were 5 grade II and 7 grade III GCTs in this series. There were 2 cases of recurrent GCT initially treated with extended curettage with recurrences detected at 14 and 17 months. Of the remaining 10 cases, 3 were confirmed on biopsy preoperatively and rest underwent frozen section at the time of surgery. None of the cases had a pathological fracture or metastatic disease at presentation. Mean follow up duration in this series was 5.8 years (8.2-3.7 years).

Mean grip strength of involved side as percentage of normal side was 71% (42-86%) and the actual mean value for operated side was 29 kg as compared to 40 kg for contralateral normal side. The average range of movements were 52° (35°-75°) forearm supination, 37° (20°-60°) forearm pronation,42° (20°-70°) of wrist palmerflexion, 31° (10°-55°) of wrist dorsiflexion with combined movements of 162° (80°-200°). Overall, 64% (29-78%) of combined range of movements were preserved on involved side as compared to contralateral normal side. Revised musculoskeletal tumor society score averaged at 91.38% (76.67-93.33%) with 5 excellent, 4 good and 3 satisfactory results. No patient was dissatisfied as far as shape of the wrist/cosmesis was concerned.

There were no major complications related to the procedure. One patient developed superficial infection at operative site which settled after a prolonged course of antibiotics for 6 weeks. There were no graft related complications like graft resorbtion or graft fracture. There were 3 cases of wrist subluxation, including one in the patient who had superficial infection in postoperative period, and all of them had some pain and functional impairment with moderate activity. There was one case of soft tissue recurrence of GCT after 2 years which was treated with excision of mass and patient has not shown any further signs of recurrence after a follow up of 4 years. Rest of 11 cases had not shown any sign of recurrence at last follow up. Two patients had non union for which iliac crest bone grafting was repeated at 10 and 13 months and graft ultimately united at 14 and 16 months respectively. There was no radiological or intraoperative evidence of inadequate fixation or significant gap at fracture sites and implant was retained in both cases. Excluding these two cases, average time for union at fibuloradial junction was 27 weeks (14-37 weeks) and the overall time for union in 12 patients averaged 33 weeks (14-69 weeks).

Weakness of extensor hallucis longus was a frequent but temporary and not troublesome complication at donor site occurring in 9 patients and recovering within 2 months in all patients. There were no cases of peroneal nerve palsy or ligamentous insufficiency related to donor site.

## Discussion

The clinical behaviour of GCT is unrelated to histological or radiological grading [3,5] and thus the decision to either salvage or excise the tumorous bone is based on ability to achieve stability and function whatever may be the means used [24]. The indications for en bloc resection would thus include pathological fractures, extensive bone involvement with large soft tissue involvement and collapse of articular surface [16,24]. Frankly malignant and recurrent tumor may also undergo en block excision or amputation.

Management of GCT of distal radius which represents around 10% of GCTs involving bone [2,5] is particularly challenging due to invariably extensive destruction of bone and an aggressive clinical behaviour [3,4]. En bloc excision is a reliable procedure in terms of lower recurrence rates but creates a bony defect and thus is reserved for large lesions with extended curettage being the treatment of choice for smaller grade I tumors [2,3,8,15,16].

Ipsilateral fibular nonvascularised autograft reconstruction of the large defect created after resection of distal radius offers many advantages over other procedures. It has low donor site morbidity, if any, with predictable and satisfactory functional results and is relatively free of major complications although minor complications occur frequently[8,9,12,16,18-22,25-27]

We achieved better or similar functional results compared to previously published series with average grip strength of 71%(42-86%) of contralateral normal side and average combined movements of 64% (29-78%). Of particular note was relatively well preserved forearm supination and pronation movements which are most important in terms of functional ability. Average time for union at host graft junction was 33 weeks(14-69 weeks) in this series which is comparable to that reported by other authors where rigid fixation and primary bone grafting was used [21,27]. Although it has been suggested that a vascularised fibula has advantage of earlier union, several authors have reported similar union time for non vascularised fibular graft if rigid fixation and primary bone grafting is used [10,11,16,17] and similar observation has been made in this series. Site of entry of nutrient artery to fibula is variable amongst general population which sometimes necessitates harvesting of a longer graft than required [10] and it has been further suggested that use of rigid fixation with plate and screws, which is the norm these days may jeopardize the vascularity of fibular graft forcing it to act essentially as a nonvascular graft [16]. Moreover, long surgical time and unavailability of required expertise of a vascular surgeon are further drawbacks which preclude the use of vascularised fibular grafting at many centres.

Most frequent complication in our series was wrist subluxation which occurred in 3 cases. These cases were managed with removable wrist splint worn during night and as needed due to pain during the daytime. This has been a frequently reported complication in other previously published series as well [16,18,19,26,27]. In a report of 24 cases, Saikia et al [27] reported 10 cases of subluxation, 6 of which were asymptomatic. Aithal et al [19] reported 3 instances of subluxation amongst 30 cases after an average follow up of 8.5 years. Maruthainar et al [18] also reported 4 cases (n = 13) of wrist subluxation.

In our series, another significant complication was non union in two of our patients which was treated with bone grafting. Nevertheless, we eventually achieved union in both these cases. Delayed union or non union has also been frequently reported by many authors (table 1). Perhaps, the use of primary bone grafting at graft radius junction has decreased the incidence of non union in more recent series [16,21,27].

We also had one case of superficial infection which was treated with prolonged course of antibiotics. Furthermore, we had one case of soft tissue recurrence which was managed with a repeat surgery and remained tumor free at latest follow up of 4 years. Overall complications were seen 50% of the patients in our series (6 patients). There were 5 excellent, 4 good and 3 satisfactory results. A total of 3 secondary procedures were performed. All patients were satisfied with the results as regards to the shape and cosmetic result of surgery.

Due to relative rarity of this tumor, there have been few published studies evaluating results of non vascularised fibular autograft for distal radial resection. Table 3 reviews all significant series on this subject (having a minimum of 5 cases) and summarises their salient features.

**Table 3:** Literature review of case series (with a minimum of 5 patients) regarding the management of GCT of distal radius.

Authors	**No**.	Type of procedure	Diagnosis	Method of fixation	Time for union (months)	Complications	Complication rate	Follow up (years)	Results	ROM	Grip Strength	Secondary procedure
Salenius et al [12]	6	Resection arthroplasty	Chondrosarcoma (1), GCT(3), Haemangioma(2)	Screws(3), Plate (3)	NA	None	0/6	5(2-12)	All good	< 20% decrease	Sufficient for manual work at 6 months	None

Murray et al [9]	18	Arthroplasty(3), Arthrodesis (15)	GCT	IM rod with screws(3), Plate (15)	2-11(4.1), 5-19 (8.6)(7.1)	Recurrence(5), pulmonary metastasis(1), NU(5), graft fracture(3)	12/18	7.1(2- 24.2)	8 excellent, 8 good, 1 satisfactory	DF 40%(0-85), PF 30%(15-70), rest near normal	40%(2-70%)	12

Lackman et al [8]	12	Arthroplasty	GCT	Plate	NA	NU(2), Recurrence(1), graft fracture(3), Subluxation(1)	7/12	8(3-14)	6 excellent, 4 good, 2 fair	PF 21(5-45), DF 28(10-45), RD 8 (5-15), UD 16 (0-25), Pr 61(40-90), Su 27(15-65)	49%(24-88%)	4

Vander Griend et al [26]	8	Arthroplasty(2), Arthrodesis(6)	GCT	Plate	NA	subluxation(2), graft fracture(3), NU(2)	6/8	5.1(2-9)		NA	NA	5

Maruthainar et al [18]	13	Arthroplasty	GCT (10), Osteosarcoma (1), Chondrosarcoma (1), Ewing's sarcoma (1)	Plate+BG in majority	NA	subluxation(4), recurrent GCT (2)leading to amputation, metastatic disease in Ewing's(1)	8/13	4.2 (2.2-7.5)		PF 16(5-30), DF 22(0-60), RD 11 (0-26), UD 14 (0-31), Pr 66(30-90), Su 52(0-90)	57%	3

Aithal et al [19]	30	Arthroplasty	GCT	Screws(3), Rush nail(1), Plate(26)	4-6.5(5.2)	Recurrence(10) leading to 4 amputations, NU(3), infection(1), subluxation(3)	14/30	8.5(1.5-25.5)	11 good, 7 fair, 2 poor(excluding recurrences)	> 65% in 7, 35-64% in 7, <34% in 3, fused wrist in 3	> 65% in 11, 35-64% in 7, <34% in 2	6

Asavamongkolkul et al [20]	7	Arthroplasty	GCT	Plate	5(4-7)	radiocarpal arthritis(2)	0/7	5.8(4.2-8)	6 excellent, 1 good	DF 45°, PF 38°, RD 20°, UD 28°, Su 80°, Pr 42° (73.7%)	69%	none

Bassiony et al [21]	10	Arthroplasty	GCT	Plate+BG	7(4-12)	NU(1), graft resorbtion (1), recurrence (1)	3/10	3.9(2.5-5)	NA	100.5(60-125)	NA	3

Saikia et al [27]	24	Arthroplasty	GCT	Plate+BG	6.7(6.5-7.25)	subluxation(10; 6 asymtomatic), recurrence(1), infection(1), graft fracture(1), wrist arthrosis(2)	9/24	6.6(2-11)	6 excellent, 14 good, 4 fair	DF 50, PF 38, RD 12, UD 22, Su 52, Pr 46, (63%;52-78)	67%(58-74)	2

Chadha et al [16]	9	Arthroplasty	GCT	Plate	6	Recurrence(1), graft fracture(2), radial a. injury(1), subluxation(1), tourniquet plasy(1), graft resorbtion (1)	5/9	4.7(3.2-5.75)		DF 40°, PF 30°, Su 45°, Pr 45°	50%	4

(NU = non-union, DF = dorsiflexion, PF = palmer flexion, RD = radial deviation, UD = ulnar deviation, Pr = pronation, Su = supination)

Several authors have advocated arthrodesis rather than an arthroplasty in view of high incidence of carpal subluxation in later [9,26]. However we believe that an arthroplasty offers several advantages and should be the surgery of choice. Firstly, it preserves the wrist flexion extension which becomes restricted in an arthrodesis. Secondly, although subluxation is a common complication, it is frequently asymptomatic and doesn't preclude a favourable clinical outcome. Thirdly, it has been observed that spontaneous fusion of joint occurs in a subset of patients with arthroplasty especially those who had fibulocarpal K wire for stabilisation. Fourthly, it is known that cartilage acts as an effective barrier for GCT and denuding carpal bones of this cartilage would make them more susceptible for involvement with GCT if a recurrence were to occur which would make a further attempt at salvage surgery more complicated and difficult. Lastly, if needed due to severity of symptoms of subluxation, arthrodesis can still be easily achieved with a relatively simple procedure. We were fortunate enough as to not use this option. All of our patients tolerated the symptoms of subluxation well without need for further surgery.

Results similar to fibular grafting have been reported with allograft reconstruction by several authors [28-30]. However, this procedure always carries a risk of disease transmission, immunological reaction and infection apart from having high complication rates [28]. Moreover, lack of availability of allograft and specialised bone bank facilities may prevent its frequent use. Translocation of ulna is another procedure which has been used frequently with good results but may not give cosmetically acceptable results as there is narrowing of wrist and distal forearm giving an hourglass appearance to the limb [26,31,32]. Endoprosthetic replacement of distal radius has also been attempted by few authors but results of such procedures have not been conclusively shown to be better than other existing treatments as most instances are of either case reports or very small series with relatively short follow up [7,33,34].

## Conclusion

To conclude, we believe that although results of non vascularised fibular autograft reconstruction of distal radius show substantial loss of function as compared to normal wrist, it still gives subjective results acceptable to most patients and comparable to all other available methods of such reconstruction. The technique also carries the advantage of not requiring the facilities of bone bank or microvascular surgery. The complication rates associated with such reconstruction of distal radius are universally high but don't preclude satisfactory results. Thus, non-vascularised fibular autograft reconstruction arthroplasty of distal radius can be considered as a reasonable procedure after en bloc excision of Grade II/III GCT.

## Conflict of Interests

The authors declare that they have no competing interests.

## Authors' contributions

Dr KB and Dr VB reviewed the literature and wrote the paper. Dr RS, Dr MSD and Dr SSG were main operating surgeons in the whole series and critically reviewed the paper. Dr RS, Dr KB and Dr AKM maintained all the records of the patients and followed them. All the authors read and approved the final manuscript
